# Evaluation of a physical activity intervention for new parents: protocol paper for a randomized trial

**DOI:** 10.1186/s12889-017-4874-7

**Published:** 2017-11-09

**Authors:** Alison Quinlan, Ryan E. Rhodes, Mark R. Beauchamp, Danielle Symons Downs, Darren E. R. Warburton, Chris M. Blanchard

**Affiliations:** 1 Behavioural Medicine Laboratory, University of Victoria, 3800 Finnerty Rd., Victoria, B.C. V8P-5C2 Canada; 2 University of British Columbia, Vancouver, BC Canada; 3 The Pennsylvania State University, State College, PA USA; 4 Dalhousie University, Halifax, NS Canada

**Keywords:** New parents, Physical activity, Planning, Self-regulation

## Abstract

**Background:**

Identifying critical life transitions in people’s physical activity behaviors may illuminate the most opportune intervention apertures for chronic disease prevention. A substantive evidence base now indicates that parenthood is one of these critical transition points for physical activity decline. This study will examine whether a brief theory-based intervention can prevent a decline in physical activity among new parents over 6 months following intervention. This study protocol represents the first dyad-based physical activity initiative in the parenthood literature involving both mothers and fathers; prior research has focused on only mothers or only fathers (albeit limited), and has shown only short-term changes in physical activity.

This study will be investigating whether a theory-based physical activity intervention can maintain or improve moderate to vigorous intensity physical activity measured via accelerometry of new parents over a 6 month period following intervention compared to a control group.

**Methods:**

This study is a 6-month longitudinal randomized controlled trial. Parents are measured at baseline (2 months postpartum) with two assessment points at 6 weeks (3.5 months postpartum) and 3 months (5 months postpartum) and a final follow-up assessment at 6 months (8 months postpartum). The content of the theory-based intervention was derived from the results of our prior longitudinal trial of new parents using an adapted theory of planned behavior framework to predict changes in physical activity.

**Results:**

A total of 152 couples have been recruited to date. Sixteen couples dropped out after baseline and a total of 88 couples have completed their 6-month measures.

**Discussion:**

If the intervention proves successful, couple-based physical activity promotion efforts among parents could be a promising avenue to pursue to help mitigate the declines of physical activity levels during parenthood. These findings could inform public health materials and practitioners.

**Trial registration:**

This trial has been registered with the Clinical Trials Registry maintained by the National Library of Medicine at the National Institutes of Health on April 19, 2014. The registration ID is NCT02290808.

**Electronic supplementary material:**

The online version of this article (10.1186/s12889-017-4874-7) contains supplementary material, which is available to authorized users.

## Background

There is convincing evidence that physical activity is associated with numerous health benefits and a reduced risk of chronic disease and premature mortality [51, 69]. In addition to the physical health benefits obtained by physical activity, there are also numerous mental health benefits. These include improvements in well-being, reduction of depression and anxiety, enhancements of cognitive functioning and improvements in overall quality of life [8, 24, 45]. Despite the vast number of benefits associated with regular physical activity, the majority of adults do not meet the recommended guidelines of 150 min of moderate to vigorous activity accumulated over the course of a week [16]. Results from the Canadian Health Measures Survey showed that adults aged 18-79 yr. accumulated just 12 min per day of moderate-to-vigorous physical activity (MVPA) and that only about 1 in every 5 adults was actually achieving the recommendations [16].

There is also evidence to suggest that the onset of parenthood may contribute to a decline in physical activity for adults [7, 29, 54]. First-time parents in particular represent a population who could greatly benefit from the effects of physical activity due to parenthood being associated with decreased sleep, increased stress, anxiety and reduced mental well-being [19, 20, 40]. Thus, parents of young children may be an important population to target given the evident decline in physical activity and important roles they play with regard to their children’s physical activity. Eighty-five percent of Canadians will become parents during their lifetime and the mean age for first-time parents in Canada is 28.5 yr. [61]. The additional demands of parenthood necessitate lifestyle changes and this may compromise the personal physical activity behaviors of new parents [7, 12]. Indeed, in a recent review examining longitudinal physical activity studies parenthood emerged as a significant predictor of physical activity decline [54]. In a trial examining physical activity trends across non-parents, first-time parents and second time parents, up to 50% of adults who were regularly active drop their physical activity behaviors when they become parents and this deficit is still present after 5 years [48]. The effect was shown in both fathers and mothers, suggesting that both parents experience these declines in their physical activity behaviors, although women appear to have a steeper trajectory in MVPA decline. A study by Mailey and McAuley [34] noted that mothers have the lowest physical activity self-efficacy (i.e., perceived confidence to be active) of any group they have examined, including frail elderly and diseased populations. Clearly, parents represent an important physical activity promotion demographic.

However, there has been limited research on the effectiveness of physical activity interventions targeting parents together. The majority of studies examined the impact of parents’ physical activity on their children’s’ physical activity [6], or is focused solely on the physical activity of mothers [23], with a few studies focusing on the physical activity of fathers [39]. In a review on physical activity interventions for post-natal mothers [23], 20 intervention studies were identified that had a physical activity promotion component among mothers between 4 and 12 weeks after giving birth. Out of the seven studies that focused specifically on improving physical activity among inactive moms, six had significant effects on MVPA. In the meta-analysis portion of the review, a moderate effect was found for an increase in frequency of physical activity at post-test suggesting that interventions to improve physical activity among post-natal populations can be an efficacious avenue for physical activity promotion efforts. Still, no research has examined whether intervening on physical activity in dual parent households has promise.

Despite the limited research targeting parents together to improve their physical activity, evidence from other health domains that have intervened at the couple level have shown some promise [3, 35]. For example, in a recent review evaluating couple-based interventions, four out of seven were found to be more effective than regular care [3]. From the literature that has focused on populations with chronic conditions, couple-based interventions have been found to be more effective than individual focused interventions [35]. Furthermore, there is evidence to suggest that couples change their physical activity behavior in tandem [48], and therefore targeting the couple could provide an effective mode for intervention to help mitigate the decline in physical activity during early parenthood.

### Theoretical framework

Understanding physical activity during parenthood has typically taken a social cognitive approach, where attitudes/outcome expectations, perceived norms and perceptions of capability (perceived control/self-efficacy) are considered the antecedents of intention and intention is the key causal agent in behavior [53]. The most popular applications have included social cognitive theory [5], theory of planned behavior [1] and the transtheoretical model of behavior change [41]. Overall, the evidence has not been supportive of attitude or normative changes that can predict physical activity decline in parents with the exception of some associations between affective aspects (e.g., stress relief) and physical activity [37]. By contrast, a sense of lowered capability – often from increased child care duties and the struggle to balance occupational and domestic responsibilities – has been a reliable predictor of physical inactivity in reviews of parenthood and physical activity research [7, 53]. Self-regulatory constructs (goal setting, planning, self-monitoring) have also shown evidence as explanatory mediating mechanisms within successful interventions in new mothers [23]. From a conceptual standpoint, self-regulatory constructs are often viewed as bridge constructs between good intentions and behavior [13, 27, 46, 58] and may be useful to augment traditional social cognitive approaches such as theory of planned behavior. The blend of evidence on motivational aspects such as perceived capability and self-regulatory constructs such as planning and self-monitoring form the theoretical basis for this trial.

### Pilot research

This research builds on prior longitudinal assessments of parenthood and health behaviors [49] as well as our prior randomized controlled trial examining self-regulator strategies and physical activity among parents [52].

Our prior research has shown that couples change their physical activity behavior and motivation together and therefore our intervention targets the couple. Indeed, changes at the level of the couple showed correlations between slopes and intercepts of *r* = .62 for behavior, and *r* = .39 to *r* = .71 [48, 49] for motivational constructs which demonstrates considerable symmetry in physical activity between mothers and fathers. Current approaches that target mothers may not be as effective at sustaining changes in physical activity as including both parents. Working with both parents allows for workload negotiation, social support and collective goals to be managed together. Although we have not located any published couple-based physical activity interventions for new parents to date there is evidence in other health domains/populations that couple-based interventions are more successful than individual-level interventions [9–11]. This study examines whether a couple-based physical activity intervention helps to maintain or improve physical activity among new parents.

### Objectives

The primary research question is whether a theory-based condition (based on an adapted theory of planned behavior) can maintain or improve adherence to regular moderate/vigorous intensity physical activity among new parents when compared to those in the control condition at 6 months post intervention (8 months after the birth of their first child). We also will explore four secondary research questions including:

1. Does the theory-based condition improve motivational, health-related quality of life, and health-related fitness outcomes among new parents when compared to those in the control condition at 6 months post intervention?
2. Can group differences among new parents with regard to these motivational, behavioral, and health-related fitness outcomes be explained through a mediation model?
3. Can motivational variables predict adherence?
4. Is there a seasonal, initial physical activity status, mental health or gender difference across primary outcomes by assigned condition?

### Hypotheses

We hypothesize that physical activity will be higher for parents in the theory-based condition in comparison to parents in the control condition after controlling for possible confounds. The effect may wane over time from the initial measurement period at 2 months after the onset of parenthood but all outcomes will remain significantly higher 6 months after intervention. For the secondary research questions we hypothesize that the theory-based condition will change salient underlying motives (theory of planned behavior constructs, self-regulation constructs) for physical activity because its basis is from the key results of our prior longitudinal trial of new parents [52] and past interventions among mothers [23]. Health-related physical fitness and quality of life will also be higher for this condition in comparison to the control condition. All outcomes will remain significantly higher at 6 months post-intervention in the theory-based condition compared to the standard physical activity education group. Improvements in both groups of mothers may occur due to recovery from pregnancy, but our hypotheses should still hold. The covariance of the assigned conditions (theory-based, standard) on adherence will be explained by changes in the salient underlying motives for physical activity (i.e., manipulation check). In turn, the covariance between these salient underlying motives and health-related outcomes will be explained by physical activity adherence among conditions. The approach will test Ajzen’s [1] theory of planned behavior adapted to include self-regulatory constructs as a bridge between good intentions and behavior [46]. Affective attitude and perceived behavioral control will predict intention, intention will predict self-regulation constructs of planning and self-monitoring, and these will predict adherence across conditions. As for seasonal or gender differences we hypothesize that there will be no differences, however, this question is exploratory because there is limited research at present.

## Methods

The trial will follow the consolidated standards of reporting trials statement [57].

### Trial design

This study is a two-arm, parallel design, single blinded randomized controlled trial (RCT). Participants (mothers and fathers) are randomized to one of two groups 1) physical activity theory-based condition; or 2) standard attention control condition for 6 months duration post parenthood onset (baseline assessment at 2 months postpartum and final follow-up at 8 months postpartum). The trial is registered with the National Library of Medicine at the National Institutes of Health and was registered on April 19, 2014. The registration ID is NCT02290808. We obtained ethical approval from the University of Victoria Human Research Ethics Board and all amendments to the study went through the Human Research Ethics Board.

### Participants and recruitment procedure

Participants are common law or married couples, who reside in greater Victoria, British Columbia and who are expecting or have just had their first child and are over the age of 18 years. Single parents are no doubt an interesting group for study with physical activity, but they will be excluded from this study because the intervention is targeting the couple. Same sex parents and surrogate parents are included in the study if their baby is within four-months of birth. Our prior longitudinal studies also included same sex couples, although this accounted for less than 1% of the sample [52]. Parents are included if they participate in physical activity below or above Canadian recommended guidelines [62] (i.e., 150 min of moderate or higher intensity activity per week). While many intervention studies often seek to screen out active participants in order to create change in physical activity, new parents represent a group where 50% who were previously meeting physical activity guidelines, will no longer be active at this level [37]. Our intervention is focused on both preventing that decline and improving physical activity. As a result, we are recruiting couples who are both active or inactive. Participants are screened for physical activity readiness via the Physical Activity Readiness Questionnaire for Everyone (PAR-Q+; www.eparmedx.com) [66, 67]. Those individuals who are not ready or able to participate in moderate intensity physical activity are excluded for safety reasons. This may include complicated pregnancy, caesarean section or any previous injuries in potential participants. Mental health conditions such as depression or anxiety are not contraindications to beginning a physical activity program [55], and therefore participants are not excluded if they have a mental health condition. However, we are measuring psychological distress and mental health among participants and are examining the impact this condition may have on retention and program success.

### Recruitment

Recruitment has already started and we are continuing to recruit through several clinical and community avenues including the utilization of online platforms, print campaigns, in-person recruitment, as well as on a referral basis. Study announcements outlining the research are posted to online interest sites, where they are marketed to the target demographic. Paper advertisements are distributed systematically around the city, focusing on doctor’s offices, health centers, midwiferies, recreation centers, maternity and baby stores, and any other community organizations offering prenatal classes or programming for first-time parents. Potential participants recruited via online or print methods are invited to contact the project coordinator and research assistants associated with this project, who act as the main point of contact. In-person recruitment initiatives take place at community fairs targeting new parents such as baby fairs, health shows, and community markets. At these events, brochures outlining the research are distributed. The research assistant is also available to answer any questions about the study, as well as to speak to the importance of being physically active postpartum. The research assistant then invites interested attendees to participate in the research study. A sign-up sheet is distributed for those interested in participating (provide their name, home/cell phone number, email, expected due date). Lastly, enrolled participants are invited to refer other families that may be interested in participating.

### Procedures

After interested participants contact the research assistant and are determined to be eligible to participate in the study, (and provide an approximate date for contact 2 months post-baby) we schedule a fitness test at our lab. At the initial visit to the lab, signed informed consent is obtained from participants by the project coordinator after overviewing the requirements of the study. Participants are assigned an identification number and all data is attached to this number to ensure confidentiality. A qualified exercise professional is employed to ensure consistency of the fitness testing procedures [68] and is also present at the first initial meeting. This individual is responsible for quality control throughout the fitness-testing portion of the trial. The fitness testers are blind to the treatment conditions of the participants. Both parents are given accelerometers to wear for 1 week and instructed on how to enter information about each day’s activity in a log. Fitness testers provide a short training session on how to wear and use the accelerometers. After the 1 week wear of accelerometers, participants are randomized at a 1:1 ratio to either intervention or control group, using an online randomizer program. Participants as well as the research assistants and project coordinator are aware of the group allocations, but all fitness testers are blinded to treatment allocation. The project coordinator and research assistants have to be aware of the condition to which participants are randomized in order to deliver the appropriate materials. The project coordinator and research assistant meet with participants at their home or location of choice to go through their specific intervention materials. Two people attend the house visits for safety reasons.

After the initial 6 week intervention period, couples are given online follow-up questionnaires sent via email to complete and accelerometers are dropped off to their house to be worn for another week and then are picked up by the research assistant. Contact is made initially with a phone call by the research assistant to setup a meeting time with participants and to prime them for the emailed online questionnaire. Both groups receive a site “booster” session on the same material but the intervention group focuses on revisiting their experiences over the past six-weeks and re-set goals and redefine/problem-solve obstacles. Participants in the control group receive a booster session but this is more of a general check in. The same protocol is followed at the 3 month time period. Thus, two booster sessions (six-weeks, 3 months) are provided to all participants. At 6 months follow-up (i.e., 8 months post-baby), parents are asked to return to the lab to complete a brief questionnaire, perform the final fitness test and participate in a brief end-of-trial quantitative questionnaire and qualitative interview to evaluate the impact of the intervention and usefulness of the intervention material. To gain a better understanding of the factors associated with physical activity we conduct semi-structured interviews with parents, in order to examine both *content fidelity* (“what is done”) and *process fidelity* (“how it is done”) related to the delivery of the intervention trial [18]. Although quantitative measurement of outcomes will enable us to examine the potency of our intervention, a process evaluation (whereby participants are interviewed) is also essential to examine the *extent to which the* program is delivered and implemented as planned.

To help study retention, we offer monetary compensation in the form of grocery store gift cards ($25 per participant, increasing by $5 each assessment) across the study.

### Intervention

Couples randomized to the intervention condition receive a post-baby physical activity workbook that serves as the template for a dialogue with the research assistant for the study. The intervention booklet was informed by the Theory of Planned Behavior (TPB) concepts and preliminary results of our prior trials [42], as well as the components that have been successful in prior intervention research with mothers and couple-based health interventions [10, 11, 17, 21, 22, 34, 37]. The booklet consists of two main sections. Physical activity guidelines are presented in the introductory section of the guide in order to define what is meant by regular physical activity and set the behavioral context. The first section focuses on the benefits of post-partum physical activity on immune function, a better night’s sleep, increase in overall energy levels, control of food cravings, reduction of pain, and finally prevention and treatment of baby blues. This section is intended to target our prior findings where affective attitudes and the belief that regular physical activity can reduce stress predicted those parents who were active from those who were not. The section concludes with a brainstorming exercise for couples where they list physical activities that they have found fun in the past, activities that may be enjoyable with their new baby, and activities that they might find enjoyable to do together. This brainstormed list helps create the template for physical activity planning/problem solving in the second section by contextualizing what the participants would like to do.

The second section guides participants through the process of finding time and planning for postpartum physical activity as well as identifying barriers and strategies to meet recommended guidelines via self-regulatory approaches [28, 52, 59] both personally and as a couple. Participants brainstorm a list of potential and past barriers (and then strategies to overcome these) when setting their physical activity goals. This section is intended to target the control barriers of regular physical activity that were identified in our prior research on parents and to improve self-regulatory strategies. The content addresses social support strategies as a couple, with friends/or extended family, problem solving around bad weather, and low-cost activities as well as coping with fatigue. The section concludes with a discussion of a re-set day (often Sunday) where the couple can reorganize their physical activity goals and plans for the following week and reflect on what “worked” and what “didn’t work” from the previous week. The emphasis is placed on how the couple can support each other to overcome barriers to physical activity either by sharing responsibilities or doing more activity together. For an outline of the study materials, please see the Additional file 1 and for an outline of the consent form please see Additional file 2. The two booster sessions (6 weeks and 3 months) serve to re-open the dialogue and assess how the couple is proceeding with in-person meetings between the couple and the research assistant. The benefits of physical activity, enjoyable activities to do, and goal setting and problem solving between the participants is re-explored and alternative plans and solutions are discussed if needed.

Couples in the control group receive physical activity guidelines and verbal presentation on the importance of physical activity post-partum. More specifically, fathers in the control condition receive the Recommendations for Physical Activity for Adults recommending 150 min of activity per week in bouts of 10 min and additional recommendations and guidelines about intensity, frequency and duration as well as ways to meet the recommended physical activity guidelines through structured and unstructured, and endurance and strength activities. Mothers in the control condition receive a comparable guide, entitled *Post-partum Physical Activity Guidelines,* which has relatively similar content. Mothers are also advised to incorporate kegel, core, and strengthening exercises into their routine.

### Outcome measures

#### Primary outcome measure:

*Change in Objective and Self-reported Physical Activity* Physical activity is measured objectively for seven consecutive days using the GT1M Activity Monitor at each time period (baseline, 6 weeks, 3 months, 6 months). It is designed to ascertain normal human movement without impeding activity and has been shown to provide valid and reliable estimates of physical activity [31]. Seven days has been proposed as an appropriate number of days for wearing a physical activity monitor to reliably estimate habitual physical activity [36, 63]. This length of time also aligns with validated self-report measures such as the Godin Leisure Time Exercise Questionnaire (GLTEQ) [26] and Physical Activity Recall (PAR) [56] as well as national physical activity recommended guidelines, all of which use a 7 day reference period [62]. The activity monitor is attached to an elastic belt and worn at the waist above the left hip. Best practice recommendations for accelerometry wear time suggest choosing a length of time that is sufficient to capture habitual physical activity while not becoming overly burdensome on participants or study resources [36]. Participants are instructed to wear the monitor from when they get up in the morning to when they go to bed and for at least 10 h and to remove the monitors at night and while swimming, bathing, or showering. Participants also complete a daily log / diary that identifies when the accelerometer is removed, unusual circumstances and structured activities.

Both acceleration and step-count are obtained using the monitor. Physical activity is assessed by measuring duration (total minutes worn, total movement counts/day, total minutes of sedentary, moderate-vigorous, and vigorous activity/day), frequency (bouts of sedentary, moderate-vigorous, and vigorous activity/day), and intensity. To calculate these variables the monitor is programmed to store data at 10 s intervals on each day.

As a secondary physical activity outcome, we use a self-report measure with the modified Godin Leisure-Time Exercise Questionnaire (GLTEQ) [26] asked at all four time points. The GLTEQ contains three questions, which assess the frequency of mild, moderate, and strenuous activity performed for at least 15 min of duration during free time in a typical week. A total GLTEQ score will be calculated by adding the frequency of physical activity within the moderate, and strenuous categories. An independent evaluation of this measure found its reliability and validity to compare favourably to nine other self-report measures of exercise based on various criteria including test-retest scores, objective activity monitors, and fitness indices [30].

#### Secondary outcome measures:

Motivation for physical activity is measured using the constructs of the theory of planned behavior and self-regulation strategies. These have been validated in parent populations [49, 50]. Items will measure all components of the model (affective attitude, instrumental attitude, injunctive norm, descriptive norm, perceived control, planning) including behavioral, normative, and control beliefs developed from prior pilot work in parents [37]. Five items are used to assess affective attitude and instrumental attitude and are measured on a 5 pt. scale. The items ask parents about their beliefs regarding physical activity behaviour over the next 6 weeks (i.e. “Over the next six weeks, engaging in physical activity on a regular basis would be … 1) Extremely unenjoyable to 5) extremely enjoyable”). Three items are used to measure subjective norms on a 5-point scale ranging from strongly disagree to strongly agree. Three items measuring perceived control are used to assess participants’ confidence that they can be regularly active over the next 6 weeks on a 5-point scale (strongly disagree to strongly agree). Intentions are measured with two items with 5-point scales (i.e. “Over the next six weeks, I am motivated to be physically active on a regular basis, and 2) Over the next six weeks I am determined to be physically active on a regular basis”). Control beliefs over perceived barriers are measured by asking participants to select the importance of each factor in preventing them from participating in 150 min of moderate to vigorous physical activity per week. Possible barriers include items such as family activities, work, mood, stress etc. Participants answer on a 5-point scale from “1) Does not prevent me at all, to 5) Prevents me a great deal”). The self-regulation items have been adapted from other sources [59, 64] and included 8 items to be answered on a 5 point scale from “Never” to “Very often”. For example, questions include items such as, “Over the past 6 weeks, I kept track of my physical activity in a diary or log”, and “Over the past 6 weeks, I set short-term (daily or weekly) goals for leisure-time physical activity.”

##### Musculoskeletal fitness

Grip strength, push ups, sit & reach flexibility, partial curl-ups, vertical jump, and back extension will be measured to determine the musculoskeletal fitness of both the children and parents using the procedures established by Gledhill and Jamnik [25] Change in musculoskeletal fitness from baseline to 6 months (i.e., post-intervention) will be examined.

##### Demographics

A brief section in the baseline questionnaire assesses characteristics including age, gender, marital status, ethnicity, level of education, health background, employment information, sleep, smoking behaviour, alcohol drinking and general eating behaviours.

##### Evaluation of intervention

A brief end-of-program interview is conducted for two main purposes. The first is to gain a deeper understanding of parent’s attitudes towards physical activity and to provide them with an opportunity to expand verbally on their experiences. Questions will include asking parents to talk about how their lifestyle was before the birth of their child to afterwards, if their physical activity has changed and what barriers they faced over the duration of the study. The second part of the interview will endeavor to determine how parents perceived the intervention materials and the delivery of the materials. Fidelity questions will be guided by the key points discussed in the article by Dumas et al. [18]. For example, questions will aim to ensure the intervention was delivered as designed, that the delivery did not change throughout the study, that it was delivered with consistency and with effective communication across the duration of the study and that participants adhered to their particular condition. Six questions pertaining to intervention fidelity will be included in the interview including, “Did you feel the information provided to you in the workbook and from the meetings with the research assistant helped to increase your physical activity?”, “Did you find the meeting sessions with the research investigator useful?”, “Have you been able to incorporate the strategies provided in the workbook and the ones you brainstormed during the meeting sessions?”, “Do you have any suggestions as to how the workbook or mini counseling sessions could be more useful for helping you increase your physical activity?”, “Did you find the check-in sessions helped you both work together to come up with a plan to increase your physical activity?”, and “Did you find the information and check-in sessions helped you both to work together to increase your physical activity?”.

### Analysis strategy

Missing data will be evaluated for patterns of missingness for each psychosocial variable and behavior at all time points using the dummy coding procedures of Allison [2]. Depending on the outcome of these tests (e.g., missing at random, missing completely at random, etc.) we will initiate the appropriate missing data handling strategy. Intention to treat analyses will also be performed in addition to sensitivity analysis procedures. An assessment of covariates will also be performed. As one would expect during early parenthood many factors may contribute to inactivity which include but are not limited to child care status, leave status, baseline quality of life and other general demographics. The RCT approach aids in some equalization in the group x time effect of these factors, yet it is acknowledged that some of these confounds may affect the time effect regardless of randomisation.

To address the first objective, hierarchical linear modeling will be used in HLM 6.0. First, a Level-1 no intercept model will be specified such that a main effect will be entered for father (0 = Mother; 1 = Father), Mother (0 = Father; 1 = Mother), a Father linear trend (0 = baseline; 1 = 6 months), and a Mother linear trend with all coefficients set to random. In this model, the main effects for the fathers and mothers’ intercepts represent their respective baseline minutes of MVPA, whereas the linear trends represent the change in MVPA (or not) over the 6-month interval. At Level-2, cross-level interactions will be added such that condition (0 = control; 1 = intervention) predicts the fathers’ Level-1 intercept / slope and the mothers’ Level-1 intercept / slope. These potential interactions will determine whether baseline MVPA is similar between conditions and whether the potential change in MVPA across time is similar between conditions accounting for the couple variation. Second, the fathers vs. mothers’ coefficients will be statistically compared using the multivariate hypothesis testing procedure (e.g., to determine if the magnitude of change in the minutes of MVPA is the same for fathers and mothers). Third, the correlations among the fathers and mothers’ intercepts and slopes will be examined to determine, for example, whether the mothers’ baseline minutes of MVPA are significantly associated with their own change in MVPA and / or their fathers’ change in MVPA (and vice versa).

To address the second and third objectives / hypotheses concerning the motivational, health-related quality of life, and health-related fitness outcomes, the same analytical approach as outlined in relation to objective 1 will be used when examining the variables as outcomes. However, to examine whether the Theory of Planned Behavior (TPB) variables explain the potential MVPA differences between conditions (i.e., whether the TPB variables mediate the condition/MVPA relationship), the appropriate Level-1 mediation analysis approach [32, 33] will be used treating the TPB variables as time varying covariates [60] while accounting for the couple variation. Finally, the fourth objective / hypothesis (i.e., whether season, gender) potentially moderate the condition / outcome relationships) will be examined by including the time invariant covariates (e.g., gender) at level-2 of the hierarchical model (i.e., by creating cross-level interactions to predict the fathers/wives’ intercepts and slopes at Level-1) and the time varying covariates (e.g., motivation variables, season) at Level-1 of the model. The primary investigator and project coordinator will have access to the final trial data set.

### Justification of sample size

It is recognized that two analytical approaches can be utilized in longitudinal couple studies within hierarchical linear modeling. First, a 3-level model can be created where repeated assessments (Level-1) are nested within the individual (Level-2) that are nested within the couple (Level-3) [4]. However, we have chosen to utilize the more common approach, which is to nest the individual repeated assessments (Level-1) within the couple (Level-2) ([4]; S. [43]). Therefore, we used the OpDes Program for power estimation of hierarchical linear models (S. W. [44]) to calculate the sample size needed for our analyses. Specifically, with a frequency of 4 measurement occasions, a duration of 6 months, within-person variance of 1.0, a growth rate of 1.0, and a moderate effect size (.40), a total of 200 couples (i.e., 100 couples per condition) are needed to show a significant adherence to MVPA as measured via accelerometry. The effect size represents the low-end of findings from prior intervention research with this demographic [17, 21, 22, 34, 38], yet it is clearly in the clinically meaningful range (D.E.R. [65]). These studies showed mean increases of moderate to vigorous physical activity of 80 min per week, which is over half of the recommended weekly activity for public health [62]. Our sample size includes a potential 25% attrition rate similar to the longitudinal study (thus total recruitment *N* = 267. The attrition in the prior trial was actually 15% [48], but we sought to oversample to accommodate the active component of this experimental trial compared to the prior passive prospective design. Our over-sampling procedures account for attrition due to second pregnancy or other possible reasons for drop out such as break-up, moving away, etc. The prediction-based research will be examined by group condition as well as via the collapsed sample for mediation analyses. Considering an average of five predictor IVs (TPB model), and using a small-medium effect size (f^2^ = .10) we will have sufficient power (.80) to evaluate these predictors at an alpha of .05. Our longitudinal study also supported the use of a small-medium effect size as an appropriate criterion [47]. Finally, the evaluation of physiological outcomes of participants across time will follow a 2 (condition) × 2 (time) interaction. The proposed sample size is, therefore, more than adequate to ensure sufficient statistical power for the physiological measurements.

## Results

**Fig. 1:**
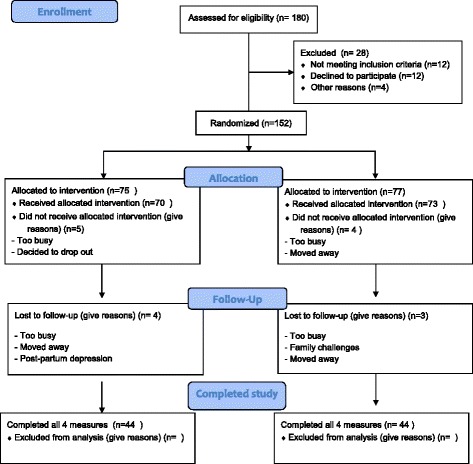
Participant Flow Diagram

The study is on-going with recruitment wrapping up in late 2017. Ethical approval was obtained, and the trial was registered with a government clinical trials database. The study has followed the Standard Protocol Items: Recommendations for Intervention Trials (SPIRIT) [14, 15] and the full checklist can be found in Additional file 3. To date a total of 152 couples have been recruited with 88 couples who have completed all four measures. Sixteen couples have dropped out after baseline testing due to a variety of reasons including not enough time, moving, and post-partum depression. Remaining participants are expected to wrap up all measures by early 2018. Please see Fig. 1 for the participant flow chart [14, 15].

## Discussion

This protocol describes the implementation of a randomized controlled trial that utilizes motivational and self-regulation strategies to try and maintain and/or increase physical activity among new parents. Research findings could be useful in public health in providing effective strategies to new parents to help prevent the decline in physical activity that often accompanies having a newborn. Additionally, findings may help to inform future interventions aimed at increasing physical activity among new parents as well as informing public health materials for new parents. Findings from this trial will be disseminated in peer-reviewed journals and presented at academic conferences.

## Additional files

Additional file 1: Outline of intervention materials. (DOCX 14 kb)

Additional file 2: Consent form. (DOCX 23 kb)

Additional file 3: SPIRIT 2013 Checklist: Recommended items to address in a clinical trial protocol and related documents. (DOC 122 kb)
