# Supplementary material for: Evaluation of a physical activity intervention for new parents: protocol paper for a randomized trial
Source: BMC Public Health. 2017 Nov 9;17:875. doi: 10.1186/s12889-017-4874-7 (PMC5679193; doi:10.1186/s12889-017-4874-7)
Supplement: Supplementary file 1 — Outline of intervention materials. (DOCX 14 kb) [file 12889_2017_4874_MOESM1_ESM.docx]

**Appendix 1 - Outline of intervention materials**

**Part 1:**

Physical Activity Guidelines

Introduction

- Brainstorming benefits of being physically active
- What the research says on the benefits of being physically active
- Finding time for exercise
- Planning – finding enjoyable activities
- Planning tips for achieving your physical activity goals
- Barriers to being physically active and strategies to overcome
- Support

**Part 2:**

Planning for physical activity worksheets

- Habits and cues
- Behaviour and rewards
- Planning
- Weekly check in

Dry-erase calendar and pen for planning

Follow-up (tracking progress)
