# Supplementary material for: Evaluation of a physical activity intervention for new parents: protocol paper for a randomized trial
Source: BMC Public Health. 2017 Nov 9;17:875. doi: 10.1186/s12889-017-4874-7 (PMC5679193; doi:10.1186/s12889-017-4874-7)
Supplement: Supplementary file 2 — Consent form. (DOCX 23 kb) [file 12889_2017_4874_MOESM2_ESM.docx]

**Consent Form**

| [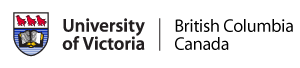](http://www.uvic.ca/%23%23) | *Participant Consent Form* |
| --- | --- |

**“First-time Parent Physical Activity Intervention”**

You are being invited to participate in a study entitled **“First-time Parent Physical Activity Intervention”** that is being conducted through the Behavioural Medicine Laboratory at the University of Victoria. The lead investigator of the study is Dr. Ryan Rhodes, and he may be contacted at 250-721-8384.

**WHAT IS THE PURPOSE OF THIS STUDY?**

The purpose of this study is to determine whether or not certain strategies administered immediately post-partum can help to improve physical activity levels among new parents in the first eight months post-partum.

**WHY IS THIS RESEARCH IMPORTANT?**

Research of this type is important because physical activity behaviour is beneficial for both physical and psychological health. Specifically, regular physical activity is associated with improvements in numerous disease states, such as cardiovascular disease, certain cancers, type 2 diabetes mellitus, depression, and overall quality of life. Despite this, recent Canadian statistics indicate that less than half of Canadian adults are active enough to reap the benefits of regular physical activity. Furthermore, there is a trend towards decreased activity levels during young adulthood, and one major factor which influences this trend is the transition to parenthood. Usually, activity levels tend to remain low rather than returning to healthy levels after this life transition. Therefore, an intervention aimed at increasing or maintaining physical activity behaviour in new parents is extremely important.

**WHAT DO PARTICIPANTS HAVE TO DO?**

If you volunteer to participate, you will be randomized to one of two groups, both groups using strategies to increase physical activity. Once it has been deemed that it is safe for you to participate in physical activity, we will schedule an appointment for you and your partner at two months post-partum, where you will come into the lab, and go through a fitness assessment with a certified personal trainer, to measure your cardiovascular fitness, musculoskeletal fitness, and body composition. We will then give you an accelerometer (which measures activity) to wear for one week, and a questionnaire to fill out. We will contact you within 1-2 weeks to come and pick up the accelerometers and questionnaires. When we come for the pick-up you will receive information on how to improve your physical activity by having a discussion with a member of our team.

This is a six month long study and we will meet with you at six weeks after the initial assessment (3.5 months post-partum) and at 3 months after the initial assessment (5 months post-partum) to have a brief ‘booster’ session to discuss how your physical activity has been going and set you and your partner up again with an accelerometer to be worn for one week. At six months (8 months post-partum) we will have a final fitness assessment, questionnaire and accelerometer wear for one week. Additionally there will be a brief 15 minute interview.

**WHAT ARE THE BENEFITS OF PARTICIPATING?** This is an exciting research project because there have been very few (if any!) interventions that have targeted physical activity in expectant parents utilizing psychology. As a participant, you will have the opportunity to increase your physical activity by utilizing psychological strategies. Furthermore, at the end of the study, you will receive the results from your fitness tests. While participating you will receive reimbursement for parking costs during your time at the lab as well as a $25 honourarium after each assessment (baseline, six weeks, three months and six months) increasing by $5 each time.

**WHAT WILL HAPPEN WITH MY DATA?** All information will be kept confidential, and data will be stored in a secure and locked location at the University of Victoria. All results will be presented and disseminated in the form of group data, and no individuals will be identified. All names and identifying characteristics will be withdrawn from the interview transcripts. The transcripts will be analyzed for common themes and will be used as group information. **Participants may withdraw without explanation or consequence at any time during the study.**

You may request further information regarding this study by contacting the lab at bml@uvic.ca or 250-472-5288; or by contacting Dr. Ryan Rhodes at rhodes@uvic.ca 721-8384. In addition to being able to contact the researcher at the above phone number and email, you may verify the ethical approval of this study, or raise any concerns you might have, by contacting the Human Research Ethics Board at the University of Victoria (250-472-4545).

Your signature below indicates that you understand the above conditions of the participation in this study, and that you have had the opportunity to have your questions answered by the researcher.

|  |  |  |  |  |
| --- | --- | --- | --- | --- |
| Name of Participant |  | Signature |  | Date |

*Please sign and return one copy to the researcher and keep one copy for yourself.*
